# Lack of knowledge and availability of diagnostic equipment could hinder the diagnosis of sarcopenia and its management

**DOI:** 10.1371/journal.pone.0185837

**Published:** 2017-10-02

**Authors:** Esmee M. Reijnierse, Marian A. E. de van der Schueren, Marijke C. Trappenburg, Marjan Doves, Carel G. M. Meskers, Andrea B. Maier

**Affiliations:** 1 Department of Internal Medicine, Section of Gerontology and Geriatrics, VU University Medical Center, Amsterdam, The Netherlands; 2 Department of Medicine and Aged Care, Royal Melbourne Hospital, The University of Melbourne, Melbourne, Australia; 3 Department of Internal Medicine, Section of Nutrition and Dietetics, VU University Medical Center, Amsterdam, The Netherlands; 4 Department of Nutrition, Sports and Health, Faculty of Health and Social Studies, HAN University of Applied Sciences, Nijmegen, The Netherlands; 5 Department of Internal Medicine, Amstelland Hospital, Amstelveen, The Netherlands; 6 Institute of Human Movement Studies, Faculty of Health Care, University of Applied Sciences Utrecht, Utrecht, The Netherlands; 7 Department of Rehabilitation Medicine, VU University Medical Center, Amsterdam, The Netherlands; 8 Department of Human Movement Sciences, MOVE Research Institute Amsterdam, Vrije Universiteit, Amsterdam, The Netherlands; Ehime University Graduate School of Medicine, JAPAN

## Abstract

**Objectives:**

Sarcopenia is an emerging clinical challenge in an ageing population and is associated with serious negative health outcomes. This study aimed to assess the current state of the art regarding the knowledge about the concept of sarcopenia and practice of the diagnostic strategy and management of sarcopenia in a cohort of Dutch healthcare professionals (physicians, physiotherapists, dietitians and others) attending a lecture cycle on sarcopenia.

**Material and methods:**

This longitudinal study included Dutch healthcare professionals (n = 223) who were asked to complete a questionnaire before, directly after and five months after (n = 80) attending a lecture cycle on the pathophysiology of sarcopenia, diagnostic strategy and management of sarcopenia, i.e. interventions and collaboration.

**Results:**

Before attendance, 69.7% of healthcare professionals stated to know the concept of sarcopenia, 21.4% indicated to know how to diagnose sarcopenia and 82.6% had treated patients with suspected sarcopenia. 47.5% used their clinical view as diagnostic strategy. Handgrip strength was the most frequently used objective diagnostic measure (33.9%). Five months after attendance, reported use of diagnostic tests was increased, i.e. handgrip strength up to 67.4%, gait speed up to 72.1% and muscle mass up to 20.9%. Bottlenecks during implementation of the diagnostic strategy were experienced by 67.1%; lack of awareness among other healthcare professionals, acquisition of equipment and time constraints to perform the diagnostic measures were reported most often. Before attendance, 36.4% stated not to consult a physiotherapists or exercise therapists (PT/ET) or dietitian for sarcopenia interventions, 10.5% consulted a PT/ET, 32.7% a dietitian and 20.5% both a PT/ET and dietitian. Five months after attendance, these percentages were 28.3%, 21.7%, 30.0% and 20.0% respectively.

**Conclusion:**

The concept of sarcopenia is familiar to most Dutch healthcare professionals but application in practice is hampered, mostly by lack of knowledge, availability of equipment, time constraints and lack of collaboration.

## Introduction

The clinical relevance of sarcopenia is increasingly being recognized and a clinical challenge in our ageing population. Sarcopenia is associated with negative health outcomes such as falls [1, 2], impaired standing balance [3], physical disability [4, 5] and mortality [6, 7]. Sarcopenia is a public health burden and entails high healthcare costs associated with hospitalization, outpatient clinic visits and home healthcare expenditure [8, 9]. According to survey data from the United States, direct costs of sarcopenia may be up to 1.5% of the total healthcare costs [9]. Prevalence rates of sarcopenia vary up to 34% in geriatric outpatients dependent on the used definition [10]. To date, no consensus definition of sarcopenia has been reached, however, most recent definitions are based on measures of muscle mass, muscle strength and gait speed [11–13].

Combined intervention of physical exercise and adequate protein intake is more effective in increasing muscle mass and muscle strength compared to either physical exercise or nutritional intervention alone [14–17]. Therefore, a multidisciplinary approach is required in which different healthcare professionals play a key role in the diagnostic strategy and management of sarcopenia. This requires common knowledge about the concept of sarcopenia, a diagnostic strategy and optimal management of sarcopenia including consultation and collaboration between diverse healthcare professionals. To date, the current knowledge and practice of healthcare professionals regarding the diagnostic strategy and management of sarcopenia is unknown. This information is highly needed to properly implement and strengthen the diagnostic strategy and management of sarcopenia in clinical practice.

The primary aim of this study was to assess the current state of knowledge about the concept of sarcopenia and the current practice of the diagnostic strategy and management of sarcopenia. Secondary aims were to assess the intentions to implement the diagnostic strategy and management of sarcopenia and to identify bottlenecks during implementation of the diagnostic strategy and management in a cohort of Dutch healthcare professionals attending the Sarcopenia Road Show, a postgraduate, multidisciplinary lecture cycle for healthcare professionals with different backgrounds (physicians, physiotherapists, dietitians and others).

## Materials and methods

### Study design

This longitudinal study included 223 medical and allied Dutch healthcare professionals attending the lecture cycle ‘Sarcopenia Road Show’. Healthcare professionals worked either in primary care, nursing homes or hospitals. Medical healthcare professionals included physicians (geriatricians, internists, internist-geriatricians, nursing home physicians, general practitioners (GP) and residents, considered as one group), nurses and GP assistants; allied healthcare professionals included physiotherapists (PT), exercise therapists (ET) (PT and ET considered as one group) and dietitians.

The Sarcopenia Road Show visited four lecture locations spread over the Netherlands (‘s Hertogenbosch, Haarlem, Dordrecht, Texel) between February 2015 and September 2015. Before and directly after the lectures, attending healthcare professionals were asked to complete a printed questionnaire. Of all attending healthcare professionals, 95% completed these questionnaires. Five months after attendance, an online questionnaire was sent by e-mail to a subgroup of 147 healthcare professionals who gave permission to be contacted at a later stage, of which n = 80 (54.4%) responded. Ethical approval was not required for this study and completion of the questionnaire was taken as consent.

### Sarcopenia Road Show

The Sarcopenia Road Show comprised three lectures and three workshops in one session with the aim to transfer knowledge about the concept of sarcopenia, diagnostic strategy and management of sarcopenia. Evidence-based lectures and workshops were developed by the authors and based on the current literature due to the absence of guidelines for sarcopenia. Lectures and workshops were presented by senior lecturers (internist-geriatrician, geriatric physiotherapist and dietician) and were focused on the pathophysiology of sarcopenia, diagnostic strategy and interventions, both from exercise and nutritional perspective. Lectures were presented in a plenary session of one and a half hour, followed by three different parallel workshops whereby each healthcare professional attended one type of workshop, focusing on either the medical, exercise or nutritional aspects of sarcopenia. To diagnose sarcopenia, the definition of the European Working Group on Sarcopenia in Older People (EWGSOP) [11] was presented, including muscle mass measured by bioelectrical impedance analysis (BIA), handgrip strength measured by a hand dynamometer and gait speed measured by the four-meter walk test at normal pace. Management aimed at increasing muscle mass and muscle strength by exercise i.e. progressive resistance training [18] requiring a PT/ET and adequate protein intake [19] as well as optimal division of protein over the day [20] requiring a dietitian. Importance of collaboration between healthcare professionals for both the diagnostic strategy and management was stressed.

### Questionnaires

Questionnaires were developed by the authors. The first questionnaire was to be completed before attendance and aimed at assessing the current knowledge and clinical practice regarding sarcopenia. The second questionnaire was to be completed directly after attendance and aimed at inquiring about intentions related to the diagnostic strategy and management. The third questionnaire was to be completed five months after attendance and aimed at assessing the level of implementation. Questions related to the current occupation, working affiliation, current state of knowledge about the concept of sarcopenia, diagnostic strategy and management of sarcopenia. The complete questionnaires are enlisted in S1 Table.

### Statistical analyses

Descriptive statistics were used to calculate numbers and percentages. Analyses were stratified by group of healthcare professionals and/or analyzed as the total group of healthcare professionals. Statistical analyses were performed using the Statistical Package for the Social Sciences 22.0 (SPSS Inc, Chicago, Illinois, USA). Visualization was performed using GraphPad Prism (version 6.3).

## Results

Table 1 shows the current occupation and working affiliation of attending healthcare professionals. Of the 223 healthcare professionals, 30.9% was physician, 14.3% nurse, 9.9% GP assistant, 37.2% PT/ET and 7.6% dietitian. In total, 45.3% of the healthcare professionals were working in primary care, 22.9% in nursing homes and 31.8% in hospitals.

**Table 1:** Current occupation and working affiliation of attending healthcare professionals (n = 223).

	Total	Medical group			Allied Health	
		Physician	Nurse	GP assistant	PT/ET	Dietitian
	n = 223	n = 69	n = 32	n = 22	n = 83	n = 17
Primary care	101 (45.3)	15 (21.7)	10 (31.3)	22 (100)	43 (51.8)	11 (64.7)
Nursing homes	51 (22.9)	9 (13.0)	3 (9.4)	NA	33 (39.8)	6 (35.3)
Hospitals	71 (31.8)	45 (65.2)	19 (59.4)	NA	7 (8.4)	0

All variables are presented as n (%).

*GP* General practitioner, *PT* physiotherapist, *ET* exercise therapist, *NA* not applicable

**Fig 1:**
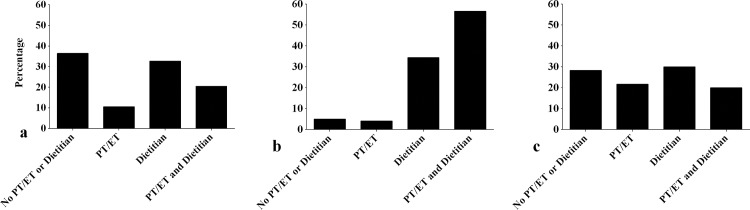
**Management of sarcopenia depicted as percentages of consulted healthcare professionals for interventions**: (a) before attendance (n = 223); (b) directly after attendance (intention to consult) (n = 223) and (c) five months after attendance (data available in n = 60). *PT* physiotherapist, *ET* exercise therapist.

Table 2 shows the current state of knowledge about the concept of sarcopenia and diagnostic strategy of healthcare professionals before and directly after attendance. Before attendance, 69.7% of the healthcare professionals stated to know the concept of sarcopenia and 82.6% had treated patients with suspected sarcopenia in the previous month before attendance. In total, 21.4% indicated to know how to formally diagnose sarcopenia. In routine clinical practice, 47.5% used their clinical view to diagnose sarcopenia. Of the healthcare professionals using diagnostic measures, handgrip strength was the most frequently used measurement (33.9%), mostly performed by PT/ET (50% of the PT/ET). PT/ET also measured gait speed most frequently (30.5% of the PT/Et) compared to other healthcare professionals. Documentation of the diagnosis of sarcopenia in clinical records was reported by 10.5% of the healthcare professionals. Fig 1A visualizes the management of sarcopenia depicted as percentages of consulted healthcare professionals for interventions for the total group of healthcare professionals. In case sarcopenia is diagnosed, 36.4% stated not to consult a PT/ET or dietitian, 10.5% consulted a PT/ET, 32.7% a dietitian and 20.5% both a PT/ET and dietitian. Results stratified by groups of healthcare professionals are shown in S2 Table. Of the medical healthcare professionals, 29.7% reported a lack of collaboration with PT/ET and 13.5% with dietitians. Of the PT/ET, 41.1% reported a lack of collaboration with medical healthcare professionals and 12.3% with dietitians. Of the dietitians, 26.7% reported a lack of collaboration with medical healthcare professionals and 33.3% with PT/ET.

**Table 2:** Current state of knowledge about the concept of sarcopenia and diagnostic strategy of healthcare professionals before and directly after attendance.

		Total	Medical group			Allied health	
			Physician	Nurse	GP assistant	PT/ET	Dietitian
		n = 223	n = 69	n = 32	n = 22	n = 83	n = 17
**Knowledge about the concept**							
Before	Knows the concept^a^	154 (69.7)	39 (57.4)	23 (71.9)	13 (59.1)	63 (76.8)	23 (71.9)
	Suspected sarcopenia^b,e^	181 (82.6)	60 (89.6)	22 (68.8)	15 (71.4)	68 (82.9)	16 (94.1)
	Knows how to diagnose^c^	46 (21.4)	17 (26.2)	4 (12.5)	1 (4.5)	18 (22.8)	6 (35.3)
Directly after	Knows how to diagnose^d^	214 (97.3)	63 (94.0)	31 (100)	21 (95.5)	82 (98.8)	17 (100)
**Diagnostic strategy–Diagnostic measures**							
Before	None^a^	83 (37.6)	18 (26.5)	13 (40.6)	15 (68.2)	34 (41.5)	3 (17.6)
	Clinical view^a^	105 (47.5)	41 (60.3)	17 (53.1)	5 (22.7)	32 (39.0)	10 (58.8)
	Nutritional status^a^	82 (37.1)	30 (44.1)	15 (46.9)	6 (27.3)	18 (22.0)	13 (76.5)
	Muscle mass^a^	20 (9.0)	6 (8.8)	1 (3.1)	0	9 (11.0)	4 (23.5)
	Handgrip strength^a^	75 (33.9)	22 (32.4)	6 (18.8)	1 (4.5)	41 (50.0)	5 (29.4)
	Gait speed^a^	43 (19.5)	15 (22.1)	3 (9.4)	0	25 (30.5)	0
**Diagnostic strategy–Diagnostic measures**							
Directly after	Intention to use muscle mass^d^	64 (29.1)	16 (23.9)	6 (19.4)	4 (18.2)	23 (27.7)	15 (88.2)
	Intention to use handgrip strength^d^	175 (79.5)	51 (76.1)	19 (61.3)	15 (68.2)	78 (94.0)	12 (70.6)
	Intention to use gait speed^d^	167 (75.9)	50 (74.6)	21 (67.7)	10 (45.5)	82 (98.8)	4 (23.5)
**Diagnostic strategy–Documentation of diagnosis**							
Before	Yes^d^	23 (10.5)	6 (8.8)	1 (3.1)	0	13 (16.0)	3 (17.6)
Directly after	Intention to do^d^	174 (80.9)	51 (78.5)	20 (64.5)	18 (81.8)	70 (87.5)	15 (88.2)

All variables are presented as n (%).

*GP* General practitioner, *PT* physiotherapist, *ET* exercise therapist

Data available in a subgroup of ^a^n = 221, ^b^n = 219, ^c^n = 215, ^d^n = 220

^e^Question was asked the following: “Have you seen patients in the previous month in which you suspected that there could be presence of sarcopenia?”

Directly after attendance, 97.3% of the healthcare professionals indicated to know how to diagnose sarcopenia (Table 2). Regarding the diagnostic strategy, 88.2% of the dietitians indicated to intend to measure muscle mass. This percentage was lower in the other groups of healthcare professionals. The intention to use handgrip strength and gait speed as diagnostic measures was the highest in PT/ET (94.0% and 98.8% respectively). Healthcare professionals stated in 80.9% to intend to document the diagnosis sarcopenia in clinical records (Table 2). Fig 1B visualizes the intended management of sarcopenia. In case sarcopenia is diagnosed, 5.0% did not intend to consult a PT/ET or dietitian, 4.1% intended to consult a PT/ET, 34.4% a dietitian and 56.6% both a PT/ET and dietitian. This did not differ between groups of healthcare professionals (S2 Table).

Of the healthcare professionals who completed the questionnaire five months after attendance, 15.0% were physician, 13.8% nurse, 10.0% GP assistant, 50.0% PT/ET and 11.3% dietitian; of whom 61.3% worked in primary care, 21.3% in nursing homes and 17.5% in hospitals. Table 3 shows the diagnostic strategy and management of sarcopenia of healthcare professionals. In total, 53.8% of the healthcare professionals indicated to have implemented the diagnostic strategy in clinical practice as suggested during the Sarcopenia Road Show. The criteria were said to be most frequently applied to older adults with mobility problems (37.2%). The median percentage of the patients screened for sarcopenia using the diagnostic strategy in the previous working week were indicated to be 0% (IQR 0–4.5). In routine clinical practice, 13.9% of the healthcare professionals indicated to use muscle mass as diagnostic measure, 50.6% handgrip strength and 54.4% gait speed. Bottlenecks during the implementation of the diagnostic strategy were experienced by 67.1%; lack of awareness among other healthcare professionals, the acquisition of equipment and time constraints to perform the diagnostic test were most often reported. Fig 1C visualizes the management of sarcopenia five months after attendance. In case sarcopenia was diagnosed, 28.3% stated not to consult a PT/ET or dietitian, 21.7% consulted a PT/ET, 30.0% a dietitian and 20.0% both a PT/ET and dietitian. A lack of collaboration was experienced by 36.8%.

**Table 3:** Diagnostic strategy and management of sarcopenia of healthcare professionals five months after attendance (n = 80).

	Total n = 80
**Diagnostic strategy**	
** Implementation of diagnostic strategy**	43 (53.8)
** Application of diagnostic strategy**	
All older adults^a^	12 (15.4)
Older adults with comorbidity^a^	18 (23.1)
Older adults with mobility problems^a^	29 (37.2)
Older adults with malnutrition^a^	22 (28.2)
Screening percentage, median [IQR]^b^	0 [0–4.5]
**Diagnostic measures**^**c**^	
No measures	56 (70.9)
Muscle mass	11 (13.9)
Handgrip strength	40 (50.6)
Gait speed	43 (54.4)
**Experienced bottlenecks**^**b**^	49 (67.1)
Lack of awareness among other healthcare professionals	23 (31.9)
Not convinced or motivated about sarcopenia	5 (6.9)
Acquisition of a device to measure muscle mass	22 (30.6)
Acquisition of handgrip strength device	8 (11.1)
No space for walking test to assess gait speed	3 (4.2)
Time constrains to perform the diagnostic tests	22 (30.6)
No funding source specific for sarcopenia	9 (12.5)
**Management–Collaboration**^**d**^	
Lack of collaboration	25 (36.8)

All variables are presented as n (%) unless indicated otherwise.

*IQR* interquartile range. Data available in a subgroup of ^a^n = 78, ^b^n = 72, ^c^n = 79, ^d^n = 68

## Discussion

This study reports on the current state of knowledge about sarcopenia, diagnostic strategy and management of sarcopenia among a cohort of Dutch healthcare professionals, attending a post graduate lecture cycle on sarcopenia. Although healthcare professionals with a specific interest in sarcopenia attended the lecture cycle, before attendance only a fifth indicated to know how to formally diagnose sarcopenia and only a third used at least one of the proposed diagnostic measures in routine clinical practice if objective tests were used (mostly: handgrip strength). Five months after attendance, approximately 50% indicated to use at least one diagnostic measure. Lack of awareness among other healthcare professionals, availability of equipment and time constraints to perform the diagnostic measures were most often reported as bottlenecks during implementation of the diagnostic strategy. For the management of sarcopenia, only one out of five healthcare professionals consulted both a PT/ET and a dietitian before attendance; this did not change after five months.

### Knowledge about the concept of sarcopenia

Although healthcare professionals stated to be familiar with the concept of sarcopenia, only a small percentage used diagnostic measures in clinical practice. Almost all healthcare professionals stated to have the intention to diagnose sarcopenia and the use of diagnostic measures had increased five months after attendance. However, hardly any patients were screened for sarcopenia in the working week prior to the five months evaluation. It could be presumed that the current state of knowledge and application of the diagnostic strategy is even much lower among healthcare professionals with no specific interest in sarcopenia. This implies that there is a major challenge in educating different healthcare professionals working in the field of ageing to create the required level of awareness and common knowledge. A survey among dietitians showed also that the term sarcopenia is used in only 12% of the dietitians [21]. To the best of our knowledge, there are no other studies describing the current knowledge and practice of sarcopenia among healthcare professionals. Educational lectures for healthcare professionals, like the Sarcopenia Road Show, aimed at transferring knowledge on the aforementioned topics, are a first step to create more awareness and knowledge among healthcare professionals, but further steps are necessary to facilitate implementation.

### Diagnostic strategy

Handgrip strength and gait speed were the most frequently used diagnostic measures before attendance. Healthcare professionals intended to use these diagnostic measures more frequently and their use had increased significantly five months after attendance. Muscle mass was least used as diagnostic measure and the intention to implement was much lower than handgrip strength and gait speed, but its use had increased five months after attendance. The acquisition of a device to measure muscle mass was one of the most reported bottlenecks. Clearly, financial aspects such as the acquisition of even a relatively cheap bioelectrical impedance analysis (BIA) device, creates huge barriers for implementation. Upon implementing the diagnostic measures for sarcopenia, healthcare professionals reported different bottlenecks; lack of awareness among other healthcare professionals, availability of equipment and time constraints of diagnostic measures were most often reported. Anticipating on these experienced bottlenecks is an important step to make the implementation of the diagnostic strategy more effective and eventually to improve care in older adults with sarcopenia. Note that only 80 healthcare professionals completed the questionnaire five months after attending and this subgroup could have over- or underestimated the results.

### Management of sarcopenia

The optimal treatment of sarcopenia requires a combined physical and nutritional intervention [14–17]. Before attendance, the combined consultation of a PT/ET and a dietitian was reported by one out of five healthcare professionals and this had not changed five months after attendance while half of the healthcare professionals had the intention to consult a PT/ET and a dietitian. This result indicates a gap between clinical practice and research which can be explained by the experienced bottlenecks also hindering the implementation of the management of sarcopenia, but probably also by organizational aspects such as availability, knowing where to find other healthcare professionals, and reimbursement strategies. Ideally, there should be a collaborative triangle between the physician, PT/ET and dietitian to diagnose and manage sarcopenia. However, this ideal collaboration was often absent; a lack of collaboration was experienced between the medical healthcare professionals and allied healthcare professionals before attendance and approximately a third of the healthcare professionals experienced a lack of collaboration five months after attendance.

### Implementation

Effective implementation of the diagnostic strategy and management of sarcopenia in daily practices requires many factors such as acquisition of diagnostic measurement devices, re-organization of care, collaboration between healthcare professionals, perceived needs and benefits of innovation and organizational factors [22, 23]. This study has highlighted some bottlenecks that were experienced in the implementation phase. Finally, for an effective implementation, all potential bottlenecks should be addressed in each phase of the implementation. Furthermore, a funding source specific for sarcopenia recognized by health insurance companies and the development of national and international guidelines by different professionals associations would be helpful for the implementation. Sarcopenia is recently recognized as an independent condition by the International Classification of Disease, Tenth Revision, Clinical Modification (ICD-10-CM) [24]. This will have advantages for both research and clinical practice such as the improvement of diagnosis and management, increasing awareness among other healthcare professionals and access to more epidemiological data regarding sarcopenia.

### Strengths and limitations

This study is, to the best of our knowledge, the first study assessing the current state of knowledge about the concept of sarcopenia, diagnostic strategy and management of sarcopenia among healthcare professionals with different working affiliations. Another strength is the specially developed multidisciplinary lecture cycle based on the translation from recent evidence into clinical practice. Selection bias is likely because the included healthcare professionals were the ones most interested and motivated to attend a postgraduate program. In addition, the healthcare professionals who responded to the questionnaire five months after attendance were probably the most motivated ones, in comparison to the non-responders. Other limitations of the study are the relative small group of dietitians while dietitians play an important role in the diagnostic strategy and management of sarcopenia. A final limitation is the use of questionnaires, which may have led to possible socially desirable responding.

## Conclusion

The concept of sarcopenia is familiar to most Dutch healthcare professionals but application in practice is hampered, mostly by lack of formal knowledge, availability of equipment and time constraints. For the management of sarcopenia, collaboration between healthcare professionals should be improved. Educational lectures regarding sarcopenia could be a first step to create more awareness among healthcare professionals, but more steps are required for successful implementation.

## Supporting information

S1 Table Complete questionnaires before, directly after and five months after attendance. (DOCX)

S2 Table Management of sarcopenia depicted as consulted healthcare professionals for interventions before and directly after attendance, total and stratified by group of healthcare professionals. (DOCX)
