# Supplementary material for: Lack of knowledge and availability of diagnostic equipment could hinder the diagnosis of sarcopenia and its management
Source: PLoS One. 2017 Oct 2;12(10):e0185837. doi: 10.1371/journal.pone.0185837 (PMC5624620; doi:10.1371/journal.pone.0185837)
Supplement: S1 Table — (DOCX) [file pone.0185837.s001.docx]

**S1 Table 1.** Complete questionnaires before, directly after and five months after attendance

| **Topic** | **Subtopics** | | | **Question** | | | **Answer options** | |
| --- | --- | --- | --- | --- | --- | --- | --- | --- |
| **Before attendance** | |  |  | | |  | |  |
| Current occupation |  | | | Q1 | What is your current position? | | Physician/Nurse/GP assistant/PT/ET/Dietitian | |
| Working affiliation |  | | | Q2 | Where do you work? | | Hospital/Nursing home/Primary care | |
| Knowledge about the concept | Knows the concept | | | Q3 | Do you know the concept of sarcopenia? | | Yes/No | |
|  | Suspected sarcopenia | | | Q4 | Have you seen patients in the previous month in which you suspected that there could be presence of sarcopenia? | | Yes/No | |
|  | Knows how to diagnose | | | Q5 | Do you know how to diagnose sarcopenia? | | Yes/No | |
| Diagnostic strategy | Diagnostic measures | | | Q6 | How do you currently diagnose sarcopenia in clinical practice? | | None/Clinical view/Nutritional status/ Muscle mass/Handgrip strength/Gait speed^a^ | |
|  | Documentation of diagnosis | | | Q7 | Do you document the diagnosis of sarcopenia in clinical records? | | Yes/No/Sometimes | |
| Management | Consulted healthcare professionals for interventions | | | Q8 | Which healthcare professionals do you consult when you have diagnosed sarcopenia? | | Intervene by themselves/Physician/Nurse/GP assistant/PT/ET/Dietitian^a^ | |

**S1 Table 1.** *(continued)*

| **Topic** | **Subtopics** | | **Question** | | | **Answer options** | |
| --- | --- | --- | --- | --- | --- | --- | --- |
|  | Lack of collaboration | | Q9 | Which healthcare professionals do you want to consult but are difficult to access, or there is a lack of collaboration? | | Physician/Nurse/GP assistant/PT/ET/Dietitian^a^ | |
| **Directly after attendance** | |  | | |  | |  |
| Knowledge about the concept | Knows how to diagnose | | Q10 | After this lecture cycle, do you know how to diagnose sarcopenia? | | Yes/No | |
| Diagnostic strategy | Diagnostic measures | | Q11 | Which diagnostic measures do you intent to use to diagnose sarcopenia? | | None/Muscle mass/Handgrip strength/Gait speed^a^ | |
|  | Documentation of diagnosis | | Q12 | Do you intent to document the diagnosis of sarcopenia in clinical records? | | Yes/No | |
| Management | Consulted healthcare professionals for interventions | | Q13 | Which healthcare professionals do you intent to consult when you have diagnosed sarcopenia? | | Intervene by themselves/Physician/Nurse/GP assistant/PT/ET/Dietitian^a^ | |
| **Five months after attendance** | | | | |  | |  |
| Diagnostic strategy | Implementation of diagnostic strategy | | Q14 | Have you implemented the diagnostic strategy for sarcopenia in clinical practice? | | Yes/No | |

**S1 Table 1.** *(continued)*

| **Topic** | **Subtopics** | **Question** | | **Answer options** |
| --- | --- | --- | --- | --- |
| Diagnostic strategy | Screening of patients using the diagnostic strategy | Q15 | In which patients do you apply the diagnostic measures to diagnose sarcopenia? | All older adults/Older adults with comorbidity/Older adults with mobility problems/Older adults with malnutrition^a^ |
|  |  | Q16 | What percentage of your patients have you screened on sarcopenia in the previous working week? | 0%-100% |
|  | Diagnostic measures | Q17 | Which diagnostic measures do you use to diagnose sarcopenia in clinical practice? | None/Muscle mass/Handgrip strength/Gait speed^a^ |
|  | Experience of bottlenecks | Q18 | Do you experience bottlenecks during the implementation of the diagnostic strategy for sarcopenia? | Yes/No |

**S1 Table 1.** *(continued)*

| **Topic** | **Subtopics** | **Question** | | **Answer options** |
| --- | --- | --- | --- | --- |
| Diagnostic strategy | Type of experienced bottlenecks | Q19 | What are the experienced bottlenecks during the implementation of the diagnostic strategy for sarcopenia? | Lack of awareness among other healthcare professionals /Not convinced or motivated about sarcopenia/Acquisition of a device to measure muscle mass/Acquisition of handgrip strength device/No space for walking test to assess gait speed/Time constrains to perform the diagnostic tests/No funding source specific for sarcopenia^a^ |
| Management | Consulted healthcare professionals for interventions | Q20 | Which healthcare professionals do you consult when you have diagnosed sarcopenia? | Intervene by themselves/Physician/Nurse/GP assistant/PT/ET/Dietitian^a^ |
|  | Lack of collaboration | Q21 | How is the collaboration between the physician, PT/ET and dietitian regarding sarcopenia? | Good collaboration/Lack of collaboration |

*Q* question, *PT* physiotherapist, *ET* exercise therapist, *GP* general practitioner. ^a^Multiple answers possible
