# Supplementary material for: Lack of knowledge and availability of diagnostic equipment could hinder the diagnosis of sarcopenia and its management
Source: PLoS One. 2017 Oct 2;12(10):e0185837. doi: 10.1371/journal.pone.0185837 (PMC5624620; doi:10.1371/journal.pone.0185837)
Supplement: S2 Table — (DOCX) [file pone.0185837.s002.docx]

**S2 Table 2.** Management of sarcopenia depicted as consulted healthcare professionals for interventions before and directly after attendance, total and stratified by group of healthcare professionals

|  |  | **Total** |  | **Medical group** | | | | |  | **Allied health** | | |
| --- | --- | --- | --- | --- | --- | --- | --- | --- | --- | --- | --- | --- |
|  |  |  |  | **Physician** |  | **Nurse** |  | **GP assistant** |  | **PT/ET** |  | **Dietitian** |
|  |  | **n=223** |  | **n=69** |  | **n=32** |  | **n=22** |  | **n=83** |  | **n=17** |
| Before attendance | No PT/ET or Dietitian^a^ | 80 (36.4) |  | 20 (29.4) |  | 10 (32.3) |  | 15 (68.2) |  | 29 (35.4) |  | 6 (35.3) |
|  | PT/ET^a^ | 23 (10.5) |  | 5 (7.4) |  | 3 (9.7) |  | 1 (4.5) |  | 14 (17.1) |  | 0 |
|  | Dietitian^a^ | 72 (32.7) |  | 33 (48.5) |  | 13 (41.9) |  | 5 (22.7) |  | 17 (20.7) |  | 4 (23.5) |
|  | PT/ET and Dietitian^a^ | 45 (20.5) |  | 10 (14.7) |  | 5 (16.1) |  | 1 (4.5) |  | 22 (26.8) |  | 7 (41.2) |
| Directly after attendance | Intention to consult no PT/ET or Dietitian^b^ | 11 (5.0) |  | 2 (2.9) |  | 2 (6.3) |  | 1 (4.5) |  | 3 (3.7) |  | 3 (17.6) |
|  | Intention to consult PT/ET^b^ | 9 (4.1) |  | 2 (2.9) |  | 3 (9.4) |  | 0 |  | 1 (1.2) |  | 3 (17.6) |
|  | Intention to consult Dietitian^b^ | 76 (34.4) |  | 33 (48.5) |  | 19 (59.4) |  | 6 (27.3) |  | 17 (20.7) |  | 1 (5.9) |
|  | Intention to consult PT/ET and Dietitian^b^ | 125 (56.6) |  | 31 (45.6) |  | 8 (25.0) |  | 15 (68.2) |  | 61 (74.4) |  | 10 (58.8) |

All variables are presented as n (%).

*GP* General practitioner, *PT* physiotherapist, *ET* exercise therapist

Data available in a subgroup of ^a^n=220; ^b^n=221
